# A Novel Inflammation-Based Stage (I Stage) in Patients with Resectable Esophageal Squamous Cell Carcinoma

**DOI:** 10.1155/2016/5396747

**Published:** 2016-04-26

**Authors:** Peng-Cheng Chen, Ji-Feng Feng

**Affiliations:** Department of Thoracic Surgery, Zhejiang Cancer Hospital, Hangzhou 310022, China

## Abstract

*Background.* Inflammation plays a key role in cancer. In the current study, we proposed a novel inflammation-based stage, named I stage, for patients with resectable esophageal squamous cell carcinoma (ESCC).* Methods*. Three hundred and twenty-three patients with resectable ESCC were enrolled in the current study. The I stage was calculated as follows: patients with high levels of C-reactive protein (CRP) (>10 mg/L), neutrophil-to-lymphocyte ratio (NLR) (>3.5), and platelet-count-to-lymphocyte ratio (PLR) (>150) were defined as I3. Patients with two, one, or no abnormal value were defined as I2, I1, or I0, respectively. The prognostic factors were evaluated by univariate and multivariate analyses.* Results.* There were 112 patients for I0, 97 patients for I1, 66 patients for I2, and 48 patients for I3, respectively. The 5-year cancer-specific survival (CSS) in patients with I0, I1, I2, and I3 was 50.0%, 30.9%, 18.2%, and 8.3%, respectively (I0 versus I1, *P* = 0.002; I1 versus I2, *P* = 0.012; I2 versus I3, *P* = 0.020). Multivariate analyses revealed that I stage was an independent prognostic factor in patients with resectable ESCC (*P* < 0.001).* Conclusion*. The inflammation-based stage (I stage) is a novel and useful predictive factor for CSS in patients with resectable ESCC.

## 1. Introduction

The cancer incidence and mortality have been increasing worldwide. Esophageal cancer (EC) is one of the most common cancers and remains the 4th leading cause of cancer death [1]. There are two major histologic types of EC: esophageal squamous cell carcinoma (ESCC) and esophageal adenocarcinoma (EAC). ESCC is the most common pathological type in China [2, 3]. However, the prognosis for patients with ESCC is still poor [3]. Therefore, assessing the prognostic factors in ESCC patients will become more and more important.

Recent reports revealed that inflammation plays an important role in cancer [4, 5]. Therefore, a series of inflammation-based biomarkers, such as C-reactive protein (CRP), neutrophil-to-lymphocyte ratio (NLR), and platelet-count-to-lymphocyte ratio (PLR), have been analysed in various cancers [6–11]. However, the prognostic values of these biomarkers in patients with ESCC remain uncertain [12–17]. In addition, most of these studies only evaluated one or two biomarkers without considering others. In the current study, therefore, we proposed a novel inflammation-based stage, named I stage (combination of CRP, NLR, and PLR), for predicting the prognosis for patients with resectable ESCC.

## 2. Patients and Methods

A retrospective analysis was conducted for patients with ESCC in our hospital from January 2005 to December 2008. Patients with ESCC were confirmed by histopathology. All patients underwent surgery with curative esophagectomy and standard lymphadenectomy. Patients who had received preoperative therapy were excluded. Patients with any form of acute infection or chronic inflammatory disease were also excluded. At last, 323 patients were enrolled in our study. In the current study, a cancer-specific survival (CSS) analysis was ascertained. The last follow-up was on 30 June 2013. This study was approved by the Ethical Committees of Zhejiang Cancer Hospital (Hangzhou, China). All patients were staged according to the 7th edition of the American Joint Committee on Cancer (AJCC) Cancer Staging [18].

Routine laboratory results (including CRP, neutrophil, lymphocyte, and platelet count) were extracted in retrospective medical records. The definitions of NLR and PLR were described as follows: NLR is neutrophil-to-lymphocyte ratio and PLR is platelet-count-to-lymphocyte ratio. The cut-off values for CRP, NLR, and PLR were 10 mg/L, 3.5, and 150 according to the previous studies [12, 13, 16, 17]. Therefore, the I stage was calculated as follows: patients with high levels of CRP (>10 mg/L), NLR (>3.5), and PLR (>150) were defined as I3. Patients with two, one, or no abnormal value were defined as I2, I1, or I0, respectively.

### 2.1. Statistical Analysis

The 5-year CSS was analysed by the Kaplan-Meier method. Univariate and multivariate Cox analyses were performed to analyse the prognostic factors. Pearson correlation analyses were performed to analyse the correlation. Receiver operating characteristic (ROC) curves were plotted to determine the accuracy of CRP, NLR, and PLR. A *P* < 0.05 was considered to be statistically significant. Statistical analyses were conducted with SPSS 17.0 (SPSS Inc., Chicago, IL, USA).

## 3. Results

**Figure 1:**
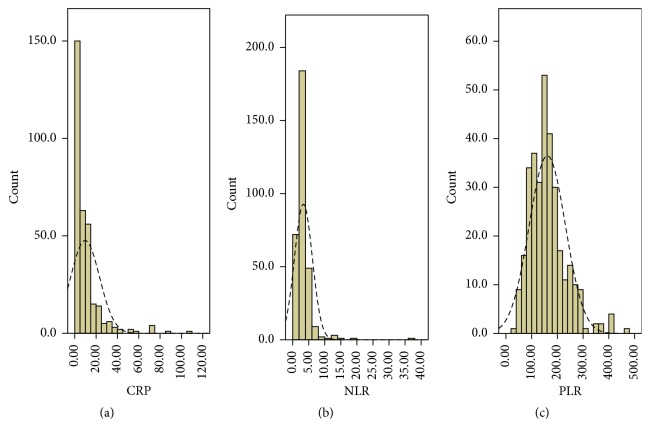
The histograms of the CRP (a), NLR (b), and PLR (c).

**Figure 2:**
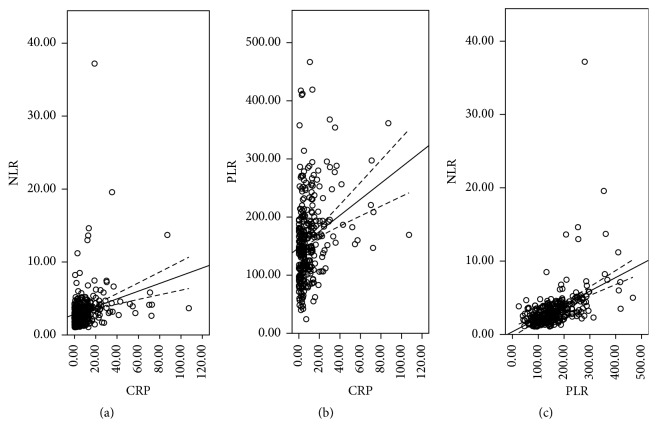
Pearson correlation analysis. Positive correlations in CRP and NLR (*r* = 0.258, *P* < 0.001; (a)), CRP and PLR (*r* = 0.265, *P* < 0.001; (b)), and NLR and PLR (*r* = 0.470, *P* < 0.001; (c)).

**Figure 3:**
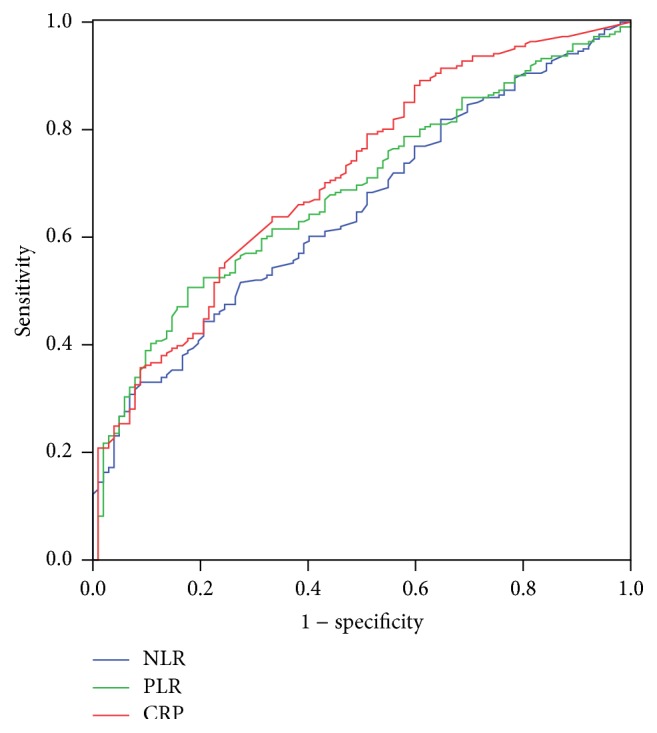
ROC curves for CSS prediction. The area under the curve (AUC) was 0.713 (95% CI: 0.653–0.772, *P* < 0.001) for CRP, 0.650 (95% CI: 0.589–0.711, *P* < 0.001) for NLR, and 0.685 (95% CI: 0.626–0.744, *P* < 0.001) for PLR.

Clinicopathologic characters were shown in Table 1. The mean CRP, NLR, and PLR were 9.7 ± 13.5 (mg/L), 3.3 ± 2.8, and 160.9 ± 70.6, respectively. The histograms of CRP, NLR, and PLR were shown in Figure 1. There were significant positive correlations in CRP and NLR (*r* = 0.258, *P* < 0.001), CRP and PLR (*r* = 0.265, *P* < 0.001), and NLR and PLR (*r* = 0.470, *P* < 0.001) (Figure 2). ROC curves for CSS prediction were shown in Figure 3. The area under the curve (AUC) was 0.713 (95% CI: 0.653–0.772, *P* < 0.001) for CRP, 0.650 (95% CI: 0.589–0.711, *P* < 0.001) for NLR, and 0.685 (95% CI: 0.626–0.744, *P* < 0.001) for PLR.

**Figure 4:**
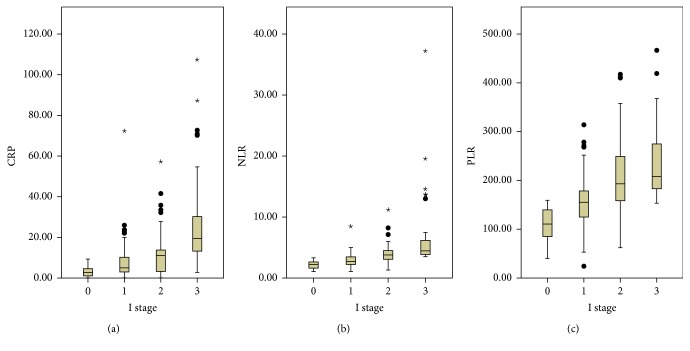
The CRP (a), NLR (b), and PLR (c) were significantly higher in patients with high I stage (*P* < 0.001). The “∗” and “•” were created by SPSS statistical software.

Of the 323 patients, 112 (34.7%) were allocated an I stage 0, 97 (30.0%) were allocated an I stage 1, 66 (20.4%) were allocated an I stage 2, and 48 (14.9%) were allocated an I stage 3, respectively. The relationships between the I stage and clinicopathological characteristics were shown in Table 2. Our study demonstrated that I stage was associated with tumor length (*P* < 0.001), perineural invasion (*P* = 0.043), T stage (*P* < 0.001), N stage (*P* < 0.001), and TNM stage (*P* < 0.001). In addition, our study revealed that CRP, NLR, and PLR were significantly higher in patients with high I stage (*P* < 0.001, Figure 4).

**Figure 5:**
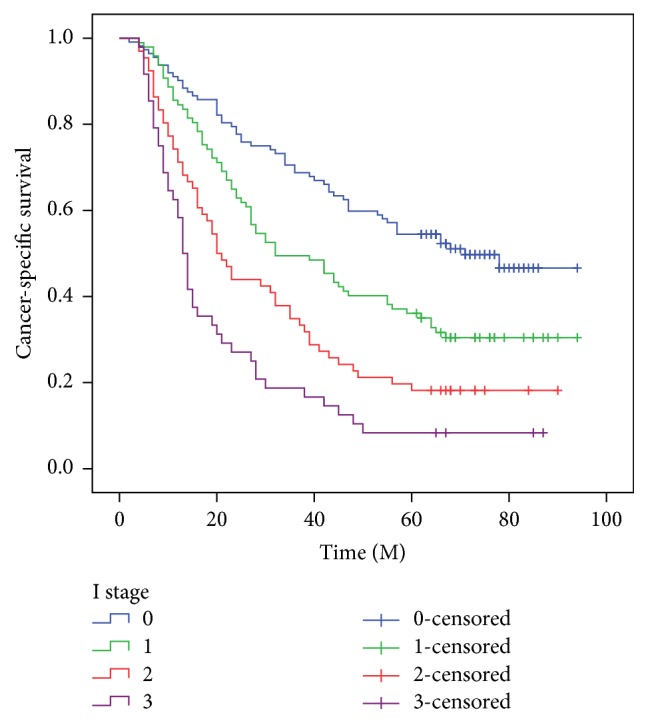
The 5-year CSS in patients with I0, I1, I2, and I3 was 50.0%, 30.9%, 18.2%, and 8.3%, respectively (*P* < 0.001) (I0 versus I1, *P* = 0.002; I1 versus I2, *P* = 0.012; I2 versus I3, *P* = 0.020).

**Figure 6:**
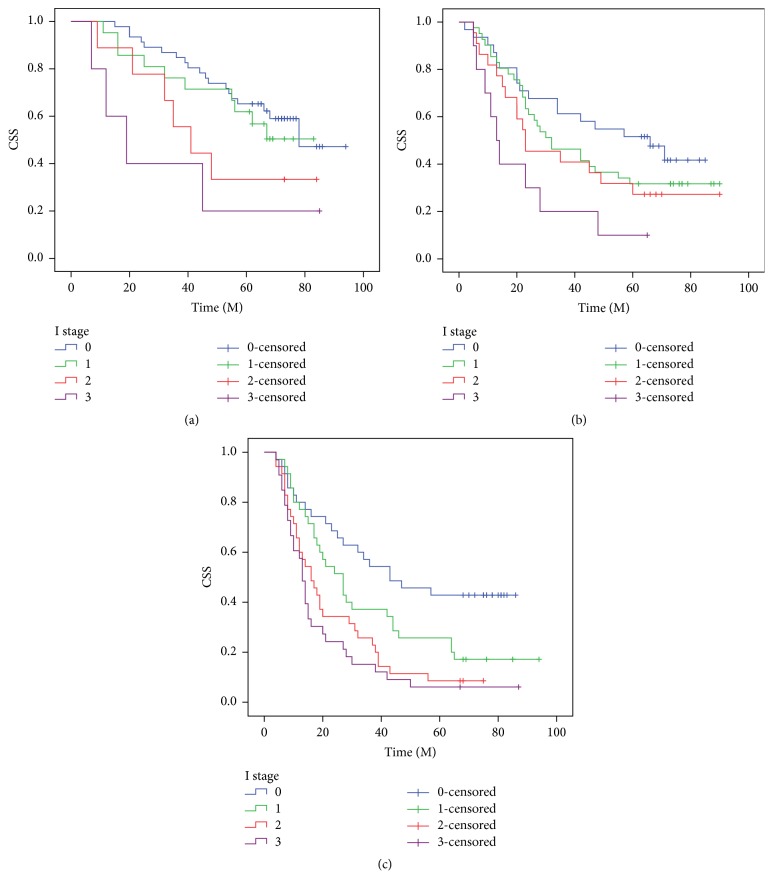
The predictive values of I stage were significant in patients based on TNM stage. TNM I stage (*P* = 0.035, (a)), TNM II stage (*P* = 0.028, (b)), and TNM III stage (*P* < 0.001, (c)).

The 5-year CSS in patients with I0, I1, I2, and I3 was 50.0%, 30.9%, 18.2%, and 8.3%, respectively (*P* < 0.001, Figure 5) (I0 versus I1, *P* = 0.002; I1 versus I2, *P* = 0.012; I2 versus I3, *P* = 0.020). In addition, our study revealed that patients with CRP (>10.0 mg/L), NLR (>3.5), or PLR (>150) were significantly associated with decreased CSS, respectively (*P* < 0.001). Then, we further stratified patients into different groups based on TNM stage. Our results demonstrated that I stage was also significantly correlated with CSS based on TNM stage (Figure 6).

Among the above variables, univariate analyses revealed that tumor length (*P* = 0.004), vessel involvement (*P* = 0.008), perineural invasion (*P* = 0.006), TNM stage (*P* < 0.001), and I stage (*P* < 0.001) were predictive of CSS (Table 3). In multivariate analyses, we demonstrated that I stage was an independent prognostic factor in patients with resectable ESCC (*P* < 0.001) (Table 4).

## 4. Discussion

In the current study, we initially proposed a novel inflammation-based prognostic system, named I stage (combination of CRP, NLR, and PLR), in patients with resectable ESCC. Our study revealed that I stage was associated with tumor length, perineural invasion, and TNM stage. In multivariate analyses, we revealed that I stage is a useful predictor of postoperative CSS in patients with resectable ESCC (*P* < 0.001).

Several hematological biomarkers have shown prognostic values in cancers. In particular, the CRP has been well validated. CRP is a representative acute-phase reactant for inflammation [19]. Recently, several previous studies have shown that CRP is associated with prognosis in several cancers, including ECs [6, 8–12]. In our study, patients with CRP ≤ 10.0 mg/L had a significantly better 5-year CSS than patients with CRP > 10.0 mg/L (39.2% versus 17.1%, *P* < 0.001). However, CRP was not an independent prognostic factor in multivariate analyses (*P* = 0.493).

The prognostic values of NLR and PLR in patients with EC remain uncertain. Several reports demonstrated that NLR is an independent prognostic factor in patients with EC [14, 15]. However, Rashid et al. [13] and Dutta et al. [16] revealed that NLR does not correlate with prognosis for patients with EC. Moreover, there have been few studies regarding PLR in EC patients. Dutta et al. [16] demonstrated that PLR does not correlate with prognosis in patients with EC. A retrospective study by Liu et al. [20] on 326 ESCC patients revealed PLR to be a potential prognostic factor. In our study, NLR and PLR were correlated with survival; however, NLR and PLN were not independent prognostic factors in multivariate analyses.

At present, the prognosis of cancer is commonly based on the TNM staging system [21, 22]. Inflammation plays an important role in cancer. Therefore, in our study, we proposed a novel inflammation-based prognostic system (I stage) in resectable ESCC patients. A significant association was found between the I stage and clinical characteristics. In multivariate analyses, we revealed that I stage is a useful predictor of postoperative CCS in patients with resectable ESCC (*P* < 0.001). It may well be that the influence of I stage on the subgroup with TNM stage is important for the understanding of its role in patients with ESCC. Our results demonstrated that I stage was also significantly correlated with CSS based on TNM stage.

Limitations should be acknowledged. Firstly, our study was a retrospective study. Secondly, we excluded patients with neoadjuvant treatment, which may have influenced the results. Neoadjuvant treatment will inevitably have an impact on the systemic inflammation. Thus, evaluation of I stage in neoadjuvant therapy does not reflect the baseline impact of systemic inflammation for ESCC patients. Therefore, larger prospective studies will need to be performed to confirm these preliminary results.

In summary, there was a significant association between the I stage (combination of CRP, NLR, and PLR) and clinical characteristics. Based on the results of the current study, we believe that I stage is a novel and useful predictive factor for CSS in patients with resectable ESCC.

## Figures and Tables

**Table 1:** Clinicopathological characteristics for patients with ESCC.

	Cases (*n*, %)
Age (years, mean ± SD)	59.1 ± 7.9
Gender	
Female	42 (13.0)
Male	281 (87.0)
Tumor length (cm, mean ± SD)	4.3 ± 1.9
Tumor location	
Upper	17 (5.3)
Middle	151 (46.7)
Lower	155 (48.0)
Differentiation	
Good	44 (13.6)
Moderate	216 (66.9)
Poor	63 (19.5)
T grade	
T1	55 (17.0)
T2	55 (17.0)
T3	179 (55.4)
T4	34 (10.6)
N stage	
N0	174 (53.9)
N1	87 (26.9)
N2	37 (11.5)
N3	25 (7.7)
TNM stage	
I	81 (25.1)
II	104 (32.2)
III	138 (42.7)
I stage	
I0	112 (34.7)
I1	97 (30.0)
I2	66 (20.4)
I3	48 (14.9)
CRP (mg/L, mean ± SD)	9.7 ± 13.5
NLR (mean ± SD)	3.3 ± 2.8
PLR (mean ± SD)	160.9 ± 70.6

**Table 2:** The relationship between I stage and clinicopathological characteristics.

	I stage 0	I stage 1	I stage 2	I stage 3	*P* value
	(*n* = 112)	(*n* = 97)	(*n* = 66)	(*n* = 48)	
Age (years)					0.817
≤60	66	58	37	25
>60	46	39	29	23
Gender					0.375
Female	18	14	5	5
Male	94	83	61	43
Tumor length (cm)					<0.001
≤3	45	31	9	4
>3	67	66	57	44
Tumor location					0.488
Upper	8	4	1	4
Middle	51	49	28	23
Lower	53	44	37	21
Vessel involvement					0.385
Negative	99	79	54	38
Positive	13	18	12	10
Perineural invasion					0.043
Negative	98	70	52	40
Positive	14	27	14	8
Differentiation					0.310
Good	17	10	12	5
Moderate	80	65	41	30
Poor	15	22	13	13
T stage					<0.001
T1	33	18	3	1
T2	23	14	11	7
T3	50	58	42	29
T4	6	7	10	11
N stage					<0.001
N0	71	55	32	16
N1	30	30	12	15
N2	6	9	12	10
N3	5	3	10	7
TNM stage					<0.001
I	46	21	9	5
II	31	41	22	10
III	35	35	35	33

**Table 3:** Univariate analyses for patients with ESCC.

	5-year CSS (%)	*P* value	HR (95% CI)	*P* value
Age (years)		0.978		0.978
≤60	30.1		1.000	
>60	33.6		0.996 (0.762–1.302)	
Gender		0.322		0.327
Female	38.1		1.000	
Male	30.6		1.227 (0.815–1.848)	
Tumor length (cm)		0.003		0.004
≤3	41.6		1.000	
>3	27.8		1.580 (1.157–2.157)	
Tumor location		0.556		0.564
Upper	41.2		1.000	
Middle	33.1		0.735 (0.385–1.404)	0.351
Lower	29.0		0.908 (0.693–1.190)	0.483
Differentiation		0.198		0.207
Good	38.6		1.000	
Moderate	31.0		1.185 (0.786–1.786)	0.417
Poor	28.6		1.504 (0.933–2.424)	0.098
Vessel involvement		0.007		0.008
Negative	34.1		1.000	
Positive	18.9		1.577 (1.129–2.202)	
Perineural invasion		0.005		0.006
Negative	35.0		1.000	
Positive	17.5		1.551 (1.135–2.119)	
TNM stage		<0.001		<0.001
I	51.9		1.000	
II	32.7		1.878 (1.269–2.780)	0.002
III	18.8		2.943 (2.039–4.248)	<0.001
I stage		<0.001		<0.001
I0	50.0		1.000	
I1	30.9		1.696 (1.189–2.420)	0.004
I2	18.2		2.676 (1.837–3.900)	<0.001
I3	8.3		4.372 (2.924–6.536)	<0.001
Adjuvant therapy		0.398		0.402
No	32.0		1.000	
Yes	30.6		1.130 (0.849–1.504)	
CRP (mg/L)		<0.001		<0.001
≤10.0	39.2		1.000	
>10.0	17.1		2.217 (1.692–2.906)	
NLR		<0.001		<0.001
≤3.5	39.0		1.000	
>3.5	17.7		1.925 (1.471–2.519)	
PLR		<0.001		<0.001
≤150	43.9		1.000	
>150	17.3		2.260 (1.729–2.955)	

**Table 4:** Multivariate analyses for patients with ESCC.

	HR (95% CI)	*P* value
Tumor length (cm)		0.603
≤3	1.000	
>3	1.075 (0.818–1.412)	
Vessel involvement		0.742
Negative	1.000	
Positive	1.060 (0.747–1.505)	
Perineural invasion		0.077
Negative	1.000	
Positive	1.341 (0.968–1.857)	
TNM stage		<0.001
I	1.000	
II	1.586 (1.048–2.400)	0.029
III	2.220 (1.456–3.384)	<0.001
I stage		<0.001
I0	1.000	
I1	1.543 (1.076–2.214)	0.018
I2	2.356 (1.602–3.466)	<0.001
I3	3.594 (2.363–5.467)	<0.001
CRP (mg/L)		0.493
≤10.0	1.000	
>10.0	1.151 (0.770–1.719)	
NLR		0.786
≤3.5	1.000	
>3.5	1.050 (0.740–1.488)	
PLR		0.065
≤150	1.000	
>150	1.440 (0.978–2.121)	
